# Society Meetings

**Published:** 1899-04

**Authors:** 


### SOCIETY MEETINGS.

The Hundred Year Club, a society for the study of longevity, has been recently organised at New York. A preliminary meeting was held March 7th, and the first regular public meeting will be held at the Waldorf-Astoria, March 23, 1899. The following programme has been arranged: Address by the chairman, Walter S. Logan; What chemistry has done for longevity in the past twenty-five years, Dr. H. W. Wiley, chemist, department of agriculture; The eclipses that shorten and the lights that lengthen the sum of human life, George W. Grover, A. M., M. D.; Recitation, Clairvoyance, Miss Lucia Morgan, of Chicago; Physical extravagance, Mrs. Almon Hensley, president society for the study of life—composed of mothers; Science and the farmer, L. H. Bailey, professor of horticulture, Cornell University; Longevity of the Hebrews, Rev. Jos. Silverman, D. D.; Song, "Freedom," Mme. Cornelia Meysenheym, court singer to the

Royal theatre in Munich, Bavaria, and Amsterdam, Holland; Discipline and longevity, Dr. Geo. M. Sternberg, Surgeon-general U. S. A.; The philosophy of rest, Mrs. E. A. Fletcher; Life insurance as bearing on longevity, Ira Mowery; "That thy days may be long," Dr. Sophia McClelland; The club, Geo. W. Smyth.

---

The National Confederation of State Medical Examining and Licensing Boards will hold its next meeting at Columbus, Ohio, June 5, 1899, beginning at 10 o'clock a. m. The following are the officers for the present year: President, William Warren Potter, Buffalo, N. Y.; vice-presidents, E. L. B. Godfrey, Camden, N. J.; William Bailey, Louisville, Ky; secretary-treasurer, A Walter Suiter, Herkimer, N. Y.; executive council, chairman, William S. Foster, Pittsburg, Pa.; Joseph M. Mathews, Louisville, Ky.; Hugh M. Taylor, Richmond, Va.; W. H. Sanders, Montgomery, Ala.; Charles K. Cole, Helena, Mont. Committee on minimum standard of requirements, chairman, N. R. Coleman, Columbus, Ohio; Gardner T. Swarts, Providence, R. I.; T. J. Happel, Trenton, Tenn.; Augustus Korndoerfer, Philadelphia, Pa.; W. R. Tipton, Las Vegas, N. M.

---

The American Medical Association will hold its fiftieth annual session at Columbus, Ohio, on Tuesday, Wednesday, Thursday and Friday June 6, 7, 8 and 9, 1899, commencing on Tuesday at 11 a. m., under the presidency of Professor Joseph McDowell Mathews, M. D., LL. D., of Louisville, Ky.

Orations will be delivered as follows: On medicine, James C. Wilson, Philadelphia; on surgery, Floyd W. McRae, Atlanta, Ga.; on state medicine, Daniel R. Brower, Chicago. Chairman committee of arrangements, Starling Loving, Columbus.

The officers of sections are: Practice of medicine, Frank Billings, Chicago, chairman; Carroll E. Edson, Denver, secretary. Surgery and anatomy, W. J. Mayo, Rochester, Minn., chairman; M. L. Harris, Chicago, secretary. Obstetrics and diseases of women, A. H. Cordier, Kansas City, Mo., chairman; W. D. Haggard, Jr., Nashville, Tenn., secretary. Materia Medica, pharmacy and therapeutics, Thomas H. Stucky, Louisville, Ky., chairman; Leon L. Solomon, Louisville, Ky., secretary. Ophthalmology, Casey A. Wood, Chicago, chairman; Charles H. Williams, Boston, secretary. Laryngology and otology, Emil Mayer, New York, chairman; Christian R. Holmes, Cincinnati, secretary. Diseases of children,

Henry E. Tuley, Louisville, Ky., chairman; L. D. Boogher, St. Louis, secretary. Physiology and dietetics, J. Weir, Jr., Owensboro, Ky., chairman; Lee Kahn, Leadville, Colo., secretary. Neurology and medical jurisprudence, Frederick Peterson, New York, chairman; Hugh T. Patrick, Chicago, secretary. Cutaneous medicine and surgery, W. T. Corlett, Cleveland, O., chairman; J. M. Blaine, Denver, Colo., secretary. State medicine, Arthur R. Reynolds, Chicago, chairman; W. P. Munn, Denver, Col., secretary. Stomatology, George V. I. Brown, Milwaukee, Wis., chairman; Eugene S. Talbot, Chicago, secretary. Wm. B. Atkinson, permanent secretary.

---

THE Buffalo Academy of Medicine held meetings during the month of March, 1899, as follows:

*Section on Surgery.*—Tuesday evening, March 7th, program: Foreign bodies in surgery, Chauncey P. Smith; discussion opened by Eugene A. Smith. Owing to the unavoidable absence of Dr. Whitbeck, of Rochester, who was to present a paper, Dr. Dowd read a short one on Urinary infection and how to prevent it.

*Section on Medicine.*—Tuesday evening March 14th, program: Differentiation of renal diseases, Thomas B. Carpenter; Acute Bright's disease, J. Henry Dowd; Chronic Bright's disease, Albert H. Woehnert; Primary nephritis, infantile, George A. Himmelsbach.

*Section on Pathology.*—Tuesday evening, March 21st, program: Tuberculous infections in the walls of the blood vessels and the production of miliary tuberculosis, H. R. Gaylord.

---

THE Western Ophthalmologic and Oto-laryngologic Association held its annual meeting at New Orleans, February 10 and 11, 1899. Owing to the unavoidable absence of the president, Dr. J. Elliott Colburn, of Chicago, the first vice-president, Dr. W. Scheppegrell, of New Orleans, presided. Two joint sessions and three sessions of the ophthalmologic and oto-laryngologic sections respectively were held and many important papers were read and discussed.

The following officers were elected for the ensuing year: Dr. W. Scheppegrell, of New Orleans, president; Dr. M. A. Goldstein, St. Louis, first vice-president; Dr. H. V. Wurdemann, of Milwaukee, second vice-president; Dr. E. C. Ellett, Memphis, Tenn., third vicepresident; Dr. F. C. Ewing, St. Louis, secretary; Dr. W. L. Dayton, of Lincoln, Neb., treasurer. St. Louis was selected for the next annual meeting.

The following names were added to the list of honorary members: Drs. George Stevens, New York; St. Clair Thompson, London, Eng.; R. Coen, Vienna, Aust.; E. J. Moure, Bordeaux, France; J. Sendziak, Warsaw, Russia; Marcel Natier, Paris, France; C. Ziem, Dantzig, Germany; A. A. Guye, Amsterdam, Holland.

The new members elected were as follows: Drs. J. A. Caldwell, McKinney, Tex.; O. Joachim, New Orleans; W. H. Baldinger, Galveston, Tex.; J. S. Mott, Kansas, City, Mo.; J. S. Lichtenberg, Kansas City, Mo.; J. W. Bettingen, St. Paul, Minn.; J. W. Chamberlin, St. Paul, Minn.; H. M. Starcky, Chicago; R. Brunson, Hot Springs, Ark; Max Thorner, Cincinnati, O.; W. Scales, Pine Bluff, Ark.; E. M. Singleton, Marshelltown, Ia. F. C. Ewing, St. Louis, Mo.

